# Laparoscopic Spleen-Preserving Distal Pancreatectomy (LSPDP) versus Open Spleen-Preserving Distal Pancreatectomy (OSPDP): A Comparative Study

**DOI:** 10.1155/2019/9367868

**Published:** 2019-07-01

**Authors:** Jing Huang, Dipesh Kumar Yadav, Chaojie Xiong, Ye Sheng, Xinhua' Zhou, Xiujun Cai

**Affiliations:** ^1^Department of Hepatobiliary and Pancreatic Surgery, Ningbo Medical Center, Medical School of Ningbo University, Ningbo, 315041 Zhejiang, China; ^2^Department of Hepatobiliary and Pancreatic Surgery, Ningbo Medical Center Lihuili Eastern Hospital, Affiliated to Taipei Medical University, Ningbo, 315041 Zhejiang, China; ^3^Department of Hepatobiliary and Pancreatic Surgery, The First Affiliated Hospital, Zhejiang University School of Medicine, Hangzhou 310009, China

## Abstract

**Objective:**

To compare outcomes between laparoscopic spleen-preserving distal pancreatectomy (LSPDP) and open spleen-preserving distal pancreatectomy (OSPDP) for treatment of benign and low-grade malignant tumors of the pancreas and evaluate feasibility and safety of LSPDP.

**Methods:**

The clinical data of 53 cases of LSPDP and 44 cases of OSPDP performed between January 2008 and August 2018 were retrospectively analyzed. The clinical outcomes between the two groups were compared.

**Results:**

There was no significant difference in preoperative data between the two groups. However, the LSPDP group had statistically significant shorter operative time (145.3±55.9 versus 184.7±33.5, P=0.03) and lesser intraoperative blood loss (150.6±180.8 versus 253.5±76.2, P=0.03) than that of the OSPDP group. Moreover, the LSPDP group also had statistically significant earlier passing of first flatus (2.2±1.4 versus 3.1±1.9, P=0.01), earlier diet intake (2.3±1.8 versus 3.4±2.0, P=0.01), and shorter hospital stay (6.2±7.2 versus 8.8±9.3, 0.04) than that of the OSPDP group. However, postoperative pancreatic fistula (P=0.64) and total postoperative complications (P=0.59) were not significantly different between the groups. The rate of pancreatic fistula and total postoperative complications occurred in 62.5% and 64.5%, respectively, in LSPDP group and, similarly, 70% and 70.0%, respectively, in OSPDP group.

**Conclusion:**

This study confirms that LSPDP is safe, feasible, and superior to OSPDP in terms of operative time, intraoperative blood loss, hospital stay, and postoperative recovery. Hence, it is worth popularizing LSPDP for benign and low-grade malignant tumors of the pancreas.

## 1. Introduction

In 1994, Ganger et al. [1] successfully performed laparoscopic pancreaticoduodenectomy for the first time. In recent years, with the advances in the laparoscopic techniques and utilization of ultrasonic scalpels, endo-GIA, and other instruments, laparoscopic distal pancreatectomy (LDP) has developed as a standard surgical procedure for a benign and malignant pancreatic tumor [2, 3]. At the same time, the rapid development of imaging technology, especially the application of endoscopic ultrasound [4], has greatly improved the detection rate of asymptomatic pancreatic tumors, providing an opportunity for early pancreatic surgery. During a distal pancreatectomy, the spleen is commonly removed for easy access and to safeguard extensive resection of lymph nodes, due to its anatomical proximity to the pancreatic tail. Notwithstanding, a concern about the immunological role of the spleen, and the idea of healthy organ preservation, has driven surgeons to avoid splenectomy at some point of time during distal pancreatectomy for benign and low-grade malignant tumors [5]. In addition, splenectomy may lead to serious postoperative complications, essentially overwhelming postsplenectomy infection (OPSI), subphrenic abscess formation, and hypercoagulability [5]. However, conservation of the spleen has been questionable to many; splenic preservation technique increases operating time, surgical risk, and postoperative complications [6]. Moreover, spleen-preserving techniques were not superior to those of splenectomy [7, 8]. In this manner, the patient's quality of life ought to be contemplated while choosing surgical strategies.

As of late, splenic preservation has progressively been recommended. Laparoscopic spleen-preserving distal pancreatectomy (LSPDP) has been supported as a standard technique for benign and low-grade malignant tumors in the distal pancreas [9]. However, the complex anatomical position and relationship of the distal pancreas with its surrounding tissues, the splenic vessels, and splenic hilum make LSPDP difficult and risky. Thus, LSPDP technique is still difficult to popularize in China. In this study, we compared 53 cases of LSPDP with 44 cases of open spleen-preserving distal pancreatectomy (OSPDP) performed in our hospital and further explore the feasibility and safety of LSPDP in clinical practice.

## 2. Materials and Methods

### 2.1. Patients

The clinical data of 53 cases of LSPDP and 44 cases of OSPDP performed at the Eastern Hospital of Li Hui Li between January 2008 and August 2018 were retrospectively analyzed and were approved by an institutional ethical board. Information gathered from the patient records was age, sex, body mass index (BMI), status of preoperative diabetes mellitus, preoperative fasting blood-glucose level, CA199 level, diameter of the tumor, histopathological report, operative time, intraoperative blood loss, postoperative first flatus time, postoperative diet intake time, postoperative hospital stay, postoperative complications, and in-hospital mortality. All patients were reviewed with preoperative imaging (CT or enhanced MRI) to precisely assess the size and location of the mass in the relationship with the splenic vasculature. Moreover, the selection of patients for this study was based on following criteria: (1) patients with a pancreatic body and tail tumor on preoperative imaging; (2) pathological report confirming benign tumor on intraoperative frozen section biopsy or postoperative histopathology; and (3) intraoperative preservation of the spleen and splenic vessels. Surgery was performed by three senior surgeons in both groups. The choice of surgical method was decided by consultation between the doctors and the patient party or according to patient wish.

### 2.2. Operative Technique and Postoperative Management 

#### 2.2.1. Surgical Procedure for LSPDP

**Figure 1:**
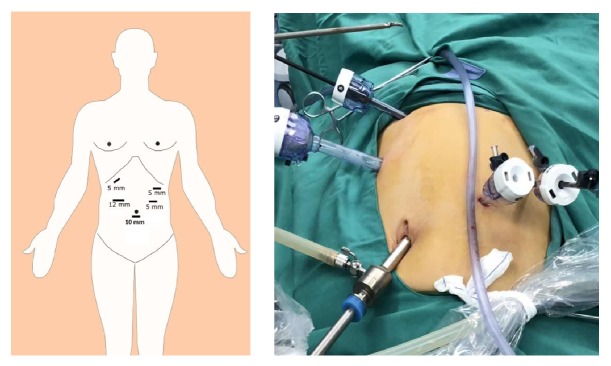
Placement of trocar for laparoscopic spleen-preserving distal pancreatectomy (LSPDP).

The patient under general endotracheal anesthesia was positioned in the supine anti-Trendelenburg position with exalted left side. A 10 mm port incision with the blade was made just below the umbilicus for the placement of 10 mm trocar as an observation hole. Moreover, the pneumoperitoneum was created with the pressure of 13-15 mm of Hg, and 10-mm trocar was inserted through the incision for the placement of a 30° telescope. Then, further four trocars were placed under the direct view of the telescope. Two trocars (12-mm and 5-mm) were placed in the right midclavicular line, slightly above the umbilical port for the operating surgeon and two 5-mm trocars in the left midclavicular line slightly below the right ports and above the umbilical port for the assistant (Figure 1).

**Figure 2:**
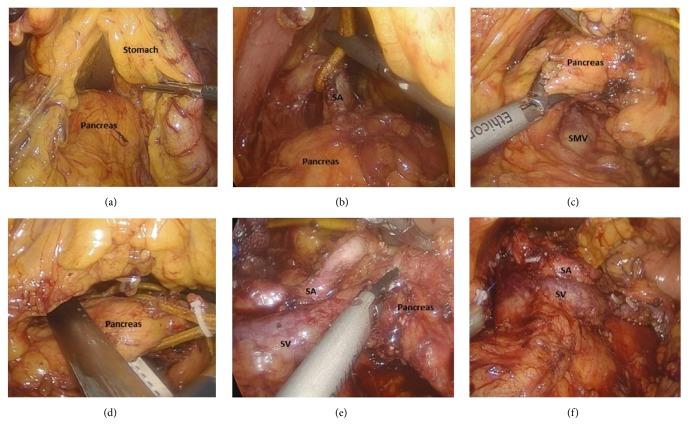
(a) Stomach is hanged superiorly and anteriorly from the abdominal wall. (b) Identification of the splenic artery and placement of the vascular loop around the splenic artery to provide counter traction. (c) Dissection of the inferior-posterior margin of the pancreas to expose superior mesenteric vein (SMV), inferior mesenteric vein (IMV), and the splenic vein. (d) The neck of pancreas is divided with a endoscopic stapler. (e) Dissection of the dorsal side of the pancreas, pulling of the distal pancreatic stump with body and tail to the left lateral side in order to free the splenic vessels from the distal pancreas. (f) Preservation of the splenic vein and artery (the body and tail of the pancreas are removed). SV: splenic vein, SA: splenic artery, and SMV: superior mesenteric vein.

Abdominal cavity was inspected for any pathology, metastasis, and to rule out any puncture to internal organs. Further, gastrocolic and gastrosplenic ligaments were dissected using a laparoscopic harmonic scalpel to expose the abdominal surface of the pancreas; the left gastroepiploic vessels and short gastric vessels were preserved. Additionally, the stomach was hanged superiorly and anteriorly from the abdominal wall, uncovering the pancreatic neck, body, and tail. After uncovering of the pancreas, intraoperative laparoscopic ultrasound was used to identify the pancreatic lesion. Moreover, with the assistance of a laparoscopic harmonic scalpel, superior-anterior margin of the pancreas was divided to separate it from the splenic artery. Firstly, the splenic artery was identified and was suspended with the help of vascular sling, and the pancreas was separated along the lower edge of the splenic artery until the tail of the pancreas by the gentle traction of the splenic artery superiorly and anteriorly (Figure 2). The gap was fully dissected and the splenic artery was completely freed. From that point, in order to separate pancreas from the retroperitoneum, the inferior border of the pancreas was dissected. Additionally, the pancreas was then pulled anteriorly and superiorly, further uncovering the superior mesenteric vein (SMV), inferior mesenteric vein (IMV), and the splenic vein located behind the pancreas in the avascular plain known as “the fusion fascia of Toldt”.

Further, the dissection was carried out longitudinally from medial to lateral in this avascular plain towards the tail of the pancreas, further divulging the splenic vein that was cautiously isolated to prevent any significant bleeding. Moreover, each branch of splenic vessels supplying the pancreas was identified and ligated using a laparoscopic harmonic scalpel or clips. Subsequently adequate surgical margins were obtained; the pancreas was proximally divided 2 cm away from the tumor applying Covidien Endo GIA Universal Straight 60-3.5 mm stapler. In addition, for the dissection of the dorsal side of the pancreas, the distal pancreatic stump with body and tail was pulled in the direction of the left lateral side, and the splenic vessels were freed from the distal pancreas with the help of an ultrasonic knife. To avoid pancreatic fistula, the pancreatic stump was sutured with polypropylene 3-0 intracorporeal interrupted sutures. Lastly, a bag was used to pull out the specimen through an enlarged umbilical port-site incision, which later was sent for histopathology. Further, warm water was employed to wash the abdominal cavity and was inspected for any bleeding; additionally, a Jackson-Pratt drain tube (JP Drain tube) was positioned close to the pancreatic stump on the left side 5 mm subcostal port-site incision.

#### 2.2.2. Surgical Procedure for OSPDP

Bilateral subcostal or upper midline incision was used to perform open surgery. Apart from the incision other approaches were similar to laparoscopic surgery, but the transection of the pancreas does not require Endo GIA stapler, the surgical blade was used for the transection of pancreatic parenchyma, and the pancreatic duct on the remnant pancreatic stump was ligated using 3-0 polypropylene continuous suture.

#### 2.2.3. Postoperative Management

The abdominal drainage tube was routinely placed after operation, and the drainage fluid amylase and serum amylase were examined after 3 days. Postoperative management for both the group of patients was done according to the enhanced recovery after surgery (ERAS) protocol, where patients were encouraged for early mobilization and early nutrition intake [10]. If the amount of peritoneal drainage was less than 10 ml at 24 hrs, the abdominal drainage tube was removed after examination with ultrasound to rule out any fluid collection in the peritoneal cavity. In addition to this, Doppler ultrasound was used during follow-up for the patency of splenic vessels.

### 2.3. Definitions

The postoperative complications as postpancreatectomy hemorrhage (PPH) [11], postoperative pancreatic fistula (POPF) [12], and delayed gastric emptying (DEG) [13] were defined in accordance with the International Study Group of Pancreatic Surgery (ISGPS). Additionally, postoperative death was defined as the death within 30-days after surgery or death during hospitalization [14].

### 2.4. Statistical Analysis

Statistical analysis was performed using SPSS 22.0 statistical software package. Continuous data were expressed as the mean ± standard deviation (SD), and t-test was used to compare the continuous variables. The categorical variables were analyzed by Chi-square tests or Fisher's exact probability test. The difference was statistically significant at P < 0.05.

## 3. Results

### 3.1. Baseline Patient Clinical Characteristics

Of the 53 patients with LSPDP, 48 cases were selected according to the criteria (3 cases were excluded from the study who were changed to open surgery for splenectomy due to intraoperative bleeding because of injury to the splenic artery or splenic vein, and another 2 cases were excluded due to malignant tumors). Out of 44 cases of OSPDP, 40 cases met the selection criteria (4 cases of malignant tumors were excluded according to the criteria). In our study, in the early years (2008-2014), 35 cases were open surgery, and only 5 cases were laparoscopic surgery. However, in later years (2014-2018) there were only 5 cases of open surgery and 43 cases of laparoscopic surgery.

The information on patients demographic characteristics of both the groups are presented in Table 1. There was no significant difference in preoperative general data between the two groups (P > 0.05). Additionally, diagnosis was confirmed by postoperative pathological report that included 5 cases of pancreatic cyst, 1 case of pancreatic pseudocyst, 8 cases of mucinous cystadenoma, 14 cases of serous cystadenoma, 9 cases of pancreatic neuroendocrine tumor (PanNET), 7 cases of solid pseudopapillary neoplasm, and 4 cases of intraductal papillary mucinous neoplasm. The postoperative pathological report of the OSPDP group included 6 cases of pancreatic cyst, 14 cases of mucinous cystadenoma, 6 cases of serous cystadenoma, 6 cases of pancreatic neuroendocrine tumor (PanNET), 3 cases of solid pseudopapillary neoplasm, 4 cases of intraductal papillary mucinous neoplasm, and 1 case of pancreatic granulomatous inflammation (Table 2).

### 3.2. Intraoperative and Postoperative Outcomes

The intraoperative and postoperative data results are presented in Table 3. Compared with intraoperative parameters, the LSPDP group had statistically significant shorter operative time (145.3±55.9 versus 184.7±33.5, P=0.03) and lesser intraoperative blood loss (150.6±180.8 versus 253.5±76.2, P=0.03) than that of the OSPDP group. Moreover, compared with postoperative parameters, the LSPDP group also had statistically significant earlier passing of first flatus (2.2±1.4 versus 3.1±1.9, P=0.01), earlier diet intake (2.3±1.8 versus 3.4±2.0, P=0.01), and shorter hospital stay (6.2±7.2 versus 8.8±9.3, 0.04) than that of the OSPDP group. However, there was no statistically significant difference in rate of postoperative pancreatic fistula (P=0.64) and total postoperative complications (P=0.59) between both the groups.

No postoperative mortality occurred in either of the groups. Pancreatic fistula occurred in 30 (62.5%) of 48 cases of LSPDP group and 28 (70%) of 40 cases of OSPDP group, respectively. Moreover, there were 27 cases of grade A pancreatic fistula and 3 cases of grade B pancreatic fistula in LSPDP group, whereas there were 25 cases with grade A pancreatic fistula and 3 cases with grade B pancreatic fistula in OSPDP group. However, there were not any cases of grade C pancreatic fistula in any of the two groups. Patients with grade A pancreatic fistula were managed by extending extubation time, adequate drainage, and antibiotic therapy. Moreover, patients with grade B pancreatic fistula were managed under CT-guided abdominal drainage. Other postoperative complications included 4 cases of intra-abdominal abscess, 2 cases of pulmonary infection, and 1 case of chyle leakage in LSPDP group and, similarly, 3 cases of intra-abdominal abscess and 3 cases of pulmonary infection in OSPDP group, respectively. However, none of the patients suffered from PPH and DEG. The patients with intra-abdominal abscess and pulmonary infection were managed with proper antimicrobial according to the culture and sensitivity results and drainage of pus for intra-abdominal abscess. Additionally, patient with chyle leakage was managed with fasting and parenteral nutrition support. In our series, splenic infarction and postoperative thrombocytosis were not seen in any of the groups.

## 4. Discussion

As of late, LDP has developed as the preferred surgical procedure for the benign and low-grade malignant tumors, with superiority of reduction in postoperative pain, reduction in wound infections, shorter hospital stay, reduction in the rate of incisional hernia, better cosmetic results, and earlier recovery after surgery than that of open distal pancreatectomy [2, 3, 15]. However, LDP for treating malignant tumor is still controversial due to its poor oncological outcome [16, 17]. Therefore, this study excluded the cases of malignant pancreatic tumors and only selected the cases of benign pancreatic diseases for comparative study, which seems to be more comparable and logical. Generally, for the simplification of the operation the spleen was removed in the course of LPD because it lies close to the pancreatic tail. And thus surgical techniques for LSPDP are more challenging for a surgeon. Nonetheless, splenectomy together with distal pancreatectomy was observed to be related to higher rates of postoperative morbidity like an OPSI, subdiaphragmatic abscess, hypercoagulability, and higher risk of cancer [5, 18, 19]. As a consequence, conservation of the spleen at the time of the LDP is suggested.

Compared with the open surgery, the laparoscopic instrument has limited flexibility and tactile feedback [20], so it is not dominant in the process of organ exposure and hemostasis. However, due to the advancement of the laparoscopic instruments, the anatomy under the endoscope is more clearly visible than the open surgery.

For the preservation of the spleen and splenic vessels during distal pancreatectomy more precise operation is required. Compared with open surgery, laparoscopic surgery has obvious advantages as mentioned earlier. For the surgeons who are more skilled in laparoscopic surgery, careful dissection around the vascular anatomy can avoid larger bleeding, and the time to achieve hemostasis can greatly be reduced. Moreover, laparoscopic surgery also saves time for opening and closing the abdomen. In general, laparoscopic surgery may take a shorter time than open surgery for surgeons who are more skilled in both the procedure. The results of this study also showed that the LSPDP group was significantly better than the OSPDP group in terms of operative time and intraoperative blood loss. Moreover, a comparison of other surgical parameters also showed that the LSPDP was superior or comparable to OSPDP in terms of surgical feasibility.

For the separation of the spleen from the tail of the pancreas, the key point should be focused on avoiding injury to the large blood vessels as much as possible. Even with the advancement of laparoscopic instruments, laparoscopic surgery still has limited means to achieve hemostasis, which are usually done with the help of clips and harmonic scalpel. In most cases, the bleeding point needs to be sutured to ensure complete hemostasis. Obviously, the placement of suture in open surgery is easier than the laparoscopic surgery. In the earlier time of LSPDP group in our series, 3 patients were changed to open surgery or spleen removal due to intraoperative bleeding. The intraoperative conversion rate was 6%. However, there were no patients in the OSPDP group who failed to preserve the spleen due to surgical reasons. The reason of conversion might be a cause of the learning curve. To correlate, in the early years of our study (2008-2014), there were only 5 cases of laparoscopic surgery. However, in later years (2014-2018) we performed 43 cases of laparoscopic surgery. This pattern suggests the growing experience and interest of the surgeon in laparoscopic surgery.

Various surgical approaches have their own pros and cons for LSPDP with the preservation of the splenic vessels; and the choice of surgical approach depends on the surgeon's experience. Nonetheless, different technique has been described in the literature such as superior-anterior approach [21], inferior-posterior approach [9], and the lateral approach [22]. Basically, superior-anterior approach and inferior-posterior approach commence dissection of pancreas from medial to lateral towards the pancreatic tail. However, superior-anterior approach focuses on the splenic artery first, whereas inferior-posterior approach focuses on the splenic vein first as a priority. On the other hand, in the lateral approach dissection of the pancreas is done from the pancreatic tail towards the pancreatic head. Lateral approach does not reveal the SMV; the free portion of the splenic vessels is comparatively shorter where there is a higher chance for the vascular injury. However, we routinely use superior-anterior approach and perform dissection from medial to lateral with respect to vascular anatomy, for following reasons: (1) because the splenic artery is relatively fixed, the anatomical relationship of the pancreas is more closely related to the splenic artery. Additionally, there is an arterial sheath around the splenic artery, and the gap between the artery and the surrounding tissue is looser than that of the vein. Moreover, the branches of the artery are less and easy to control bleeding after the separation and isolation. (2) In obese patients, the contour between pancreatic tail and splenic hilar is ambiguous due to surplus fat. (3) Anatomical variations of splenic vessels may be present in splenic hilar area and thus possibly lead to vascular injury which probably afterwards may result in surgical failure or failure to preserve the spleen and splenic vessels. However, a risk of the splenic artery injury is higher in the case of the obese patients or inflamed pancreas due to indistinctness among spleen artery and celiac trunk or due to anatomical variations of the splenic artery. Thus, the operating surgeon needs to be more careful.

The advantage of the superior-anterior approach centered to the splenic artery is that, after adequate isolation of the splenic artery, the splenic vein is separated, and both the artery and the vein are dealt separately with different measures to control any intraoperative bleeding. Otherwise, once the bleeding occurs, it is hard to locate the bleeding point because of the close proximity of the splenic artery and vein with each other, and it is often difficult to stop the bleeding. Moreover, arterial bleeding can be controlled by use of the clip. The bleeding of the small branches of the veins can be controlled by the use of laparoscopic harmonic scalpel and compression with the cotton gauze. For the bleeding of the larger branch, the area can be fully exposed and the suture and/or the clip can be placed to control the bleeding point. According to the author's experience, surgeons should try to stay away as much as possible from the trunk of the splenic vein.

In this study, postoperative recovery in the LSPDP group was significantly better than that of the OSPDP group. Specifically, the postoperative flatus time, diet intake time, and duration of hospital stay in the LSPDP group were significantly shorter than those in the OSPDP group, indicating that the overall trauma of the LSPDP group was lesser than the OSPDP group due to the smaller size of incisions and fewer bowel manipulation, by that decreasing postoperative pain and promoting the earlier ambulation and restoration of bowel function. However, pancreatic fistula still remains to be a major complication in our study after LSPDP. The result of this study showed that rate of the pancreatic fistula was 62.5% in the LSPDP group and 70.0% in the OSPDP group. Nevertheless, the difference was not statistically significant between the groups. As reported earlier in the literatures, the incidence rate of pancreatic fistula varies between 3% and 45% [12, 23–25]. Compared with the other studies, the rate of pancreatic fistula in our study is significantly higher. As per the author's experience, the author believes that there are the following reasons for a higher rate of pancreatic fistula in this study: (1) The rate of pancreatic fistula in distal pancreatectomy is indeed higher in comparison to that of pancreatoduodenectomy. (2) The data collected in this study include cases between 2008 and 2018, where almost half of the patients with the pancreatic fistula were classified according to the 2005 ISGPS consensus definition of the pancreatic fistula [26]. Compared with the 2016 ISGPS consensus definition for the pancreatic fistula [12], the definition of original grade A pancreatic is modified and no longer is considered a true pancreatic fistula, and instead it is treated as a “biochemical leak (BL)”. Most of the grade A pancreatic fistula and some grade B pancreatic fistula in this study should be classified as BL according to the new definition; that is, the actual pancreatic fistula rate should be lower than the original research data. Other surgical complications such as intra-abdominal infection, chyle leakage, and pulmonary infection were under the acceptable range.

A couple of limitations of this study should be perceived. Firstly, this study is a retrospective comparative analysis and thus potency to biases. Secondly, some clinical data are not sufficient, such as the use of analgesics, postoperative stress, immune-related indicators, medical expenses, etc., making the comparative analysis not comprehensive enough. Notwithstanding, the basic patients demographic characteristics of both the groups were similar as shown in Table 1. Likewise, the patient sample in this study is comparatively small, and thus, the results of this study should be considered with caution. Further, our study also suggests that there is a need of a multicenter randomised controlled trial to compare LSPDP and OSPDP.

In conclusion, this study confirms that LSPDP is safe, feasible, and superior to OSPDP in terms of operative time, intraoperative blood loss, hospital stay, and postoperative recovery. Hence, it is worth popularizing LSPDP for benign and low-grade malignant tumors of the pancreas.

## Figures and Tables

**Table 1:** Patient demographic characteristics of LSPDP group and OSPDP group.

Parameter	LSPDP N=48	OSPDP N=40	*t*/X^2^	p
Sex ratio, male:female	26:22	29:11	3.129	0.77

Age,years (mean± SD)	47.5±17.3	51.4±20.3	0.190	0.85

Diabetes Mellitus (n)	10	7	0.165	0.693

Fasting blood-glucose (mmol/L)	7.38±1.34	7.56±1.56	1.270	0.260

Body Mass Index (kg/m2)	23.7±1.9	24.1±2.2	1.004	0.349

CA199 (U/ml)	115.5±4.5	117.2±3.2	0.34	0.325

Diameter Of Tumor	4.1±2.0	4.3±2.1	0.81	0.659

LSPDP: laparoscopic spleen-preserving distal pancreatectomy; OSPDP: open spleen-preserving distal pancreatectomy.

**Table 2:** Pathological diagnosis.

	LSPDP N=48	OSPDP N=40
Pancreatic cyst	5	6

Pancreatic pseudocyst	1	-

Mucinous cystadenoma	8	14

Serous cystadenoma	14	6

Pancreatic neuroendocrine tumor (PanNET)	9	6

Solid pseudopapillary neoplasm	7	3

Intraductal papillary mucinous neoplasm	4	4

Pancreatic granulomatous inflammation	-	1

Total	48	40

**Table 3:** Intraoperative and postoperative data and complications.

Parameter	LSPDP N=48	OSPDP N=40	*t*/X^2^	p
Operative time (mins)	145.3±55.9	184.7±33.5	2.25	0.03
Blood loss (ml)	150.6±180.8	253.5±76.2	2.31	0.03
Post-operative first flatus time (d)	2.2±1.4	3.1±1.9	2.57	0.01
Post-operative diet intake time (d)	2.3±1.8	3.4±2.0	2.53	0.01
Complications(n)				
Chyle leakage	1	0	-	1.0^a^
Intra-abdominal abscess	4	3	-	1.0^a^
Pulmonary infection	2	3	-	0.656^a^
Pancreatic fistula [n. (%)]	30(62.5%)	28(70%)	0.546	0.640
A	27	25	0.353	0.553
B	3	3	-	1.0^a^
C	0	0	-	-
Total patients with 1 or more complications [n (%)]	31(64.5%)	28(70%)	0.290	0.590
Post-operative hospital stay(d)	6.2±7.2	8.8±9.3	2.13	0.04
In-hospital mortality [n (%)]	0	0	-	-

a: Fisher exact probability.

## Data Availability

All the data supporting the results were shown in the paper and can be available from the corresponding author.
